# Reports on the Hospitals of the United Kingdom

**Published:** 1909-10-02

**Authors:** Henry Burdett


### REPORTS
ON THE
### Hospitals of the United Kingdom.

By SIR HENRY BURDETT, K.C.B., K.C.V.O.

### I.—NORTH STAFFORDSHIRE INFIRMARY, HARTSHILL.

Where are the Governors?

Periodically every hospital, and indeed most large institutions, need to be overhauled both in regard to their constitution and rules, and in respect to their buildings and departments. The North Staffordshire Infirmary is no exception to this rule. Indeed, we should say the moment has arrived for the appointment of a special committee of governors to whom the present rules and regulations should be referred, with authority to revise them, especially in relation to the number, qualifications and duties of the honorary medical staff. This special Committee should have power to employ expert advice, and to bring up a report upon the present condition of the buildings, with plans, and estimates, so that it may be enabled to lay before the governors carefully thought out proposals for bringing this Infirmary up to date and making it in all respects a modern hospital. Recent events clearly point to the absence of sufficient supervision on the part of the governors of the internal management and general business of this Infirmary. It needs the control of a weekly Board of good and intelligent members, who would devote sufficient time and energy, each week, to its affairs. Had there been such a Board with a capable medical superintendent it would have been impossible, in our judgment, for the hospital to have fallen into its present state of inefficiency, and the recent trouble in connection with a member of the staff could not have occurred.

After a most careful inspection of the whole institution, and the fullest consideration of all the matters brought to our notice, we would venture to urge upon the governors the following changes, which seem to us essential, if the efficient working of the Infirmary is to be their first consideration. Instead of being seen, as at present, on two days a week only, there should be at least four days each week for ordinary out-patients, in addition to two days for ophthalmic cases, and one for the treatment of throat, nose and ear cases, of which there seems to be an increasing number. These changes would entail an immediate increase in the honorary medical staff, by the addition of at least two more surgeons and physicians. At the present time the hospital is understaffed, having regard to the volume of medical and surgical work which the number both of in- and out-patients at present involves. We have set forth in detail in the course of our report the grounds upon which we venture to make these recommendations, which include material reconstruction and additions to the existing buildings, especially in the out-patient and casualty departments. Two small wards for the accommodation of drunk and dying cases brought to the hospital, and where delirious cases can be nursed without harm to themselves and distress to other patients, are essential. The hospital badly needs the addition of new fittings and appliances in the out-patient and casualty departments, and in the bathrooms and lavatories, which require to be equipped with every modern convenience, so as to bring them up to date and ensure their efficient working. The fact that the hospital receives nearly half its ordinary income, £5,705 out of a total ordinary income of £12,520 from establishment subscriptions, makes the claim of the working classes upon the management paramount, where, as in the present case, the proper treatment of the patients and a due regard to their comfort, and the consideration which ought to be shown to them, are involved. Indeed it speaks volumes for the forbearance and patience of the men of the Potteries that they should have continued to subscribe so handsomely to this hospital, having regard to the present lamentable condition of its out-patient and casualty departments, and to several other matters which we will now proceed to deal with in proper order. No doubt the present state of affairs would not have been possible had there been that active and continuous supervision exercised by the governors which is so marked a feature of the majority of voluntary hospitals in this country to-day. We are quite aware that to be an efficient member of a hospital board entails labour and responsibility upon the individual, but every man who consents to join a hospital committee should be prepared to render personal service in the days of health in the cause of the sick to the full extent of the requirements of any hospital, the governors of which honour him by electing him a member of its managing body.

The Original Architect.

This hospital was rebuilt and opened in 1869, when we first visited it, and it was then remarkable as a great improvement in hospital construction. It has stood the test of time well, and parts of it, notably some of the ward floors and the embryo balconies bear striking testimony to the faithfulness and great ability of the architect Mr. Charles Lynam, who, we were glad to hear, is still alive. At that time much controversy arose, and has continued since, as to the best flooring for wards. Some of us contended that narrow boards were best. Mr. Lynam had the courage to put them down. He has his reward to-day in the excellent appearance and condition of these boards after forty years. Rufford's baths and ornamental tiles on the walls and in the corridors have worn well, and some of the former are yet in use. The chief wards were good, and are as good to-day for the treatment of disease as ever they were; so the hospital authorities have good cause to be grateful to Mr. Lynam for the excellence of much of his work.

The Wards and Buildings.

Speaking generally, the whole of the buildings on the hospital site should be reviewed by competent experts, so that the best use may be made of every portion of them. One of the wards, Ward 14, is almost underground, dark, quite unsuitable for sick people to occupy, and should be closed at once. There are no ophthalmic wards, and ophthalmic patients requiring or undergoing operations are treated in the general wards, which ought not to be. There is no separate ophthalmic theatre. The children's wards have been proved to be most insanitary, and need at once complete reconstruction, for several cases of diphtheria have occurred in them, which has led to the closing of the wards for over three months, though no steps have been taken to submit them to an exhaustive and scientific examination with a view to the removal of the existing floors, and, if necessary, the gutting of the buildings, so as to ensure that they shall be made worthy of a modern hospital, and suitable for the safe treatment of disease. The lavatory and bathrooms need enlarging, and a large verandah should be constructed for each floor, which should not be less than 10 feet wide. Another feature which shows an absence of intelligent oversight and interest on the part of the authorities is the empty room at the entrance to each ward, used formerly as a sister's bedroom, which ought to have been long ago furnished as a sitting-room. We heard something about the danger of a day-room of the kind, but such things are unthinkable in a modern, up-to-date hospital in the present day which is properly administered, where all are animated by the right spirit. We noticed that although open-air wards are a feature on the male side, no such accommodation is provided in connection with the women's wards, and it should be supplied promptly. The isolation block is a temporary iron structure, the sanitation, heating and comfort of which are deplorable. It would be cruel to think of putting any patient on any pretence into these wards as they exist to-day.

A Disgraceful Out-patient Department.

Bad as these defects are, they are nothing compared with those of the present out-patient department. The present buildings are quite unsuitable for a modern hospital, and are wholly inadequate for the work which has to be done. There is no proper system of classification of new patients, and their treatment when they attend the hospital for the first time must cause the patients just indignation. The accommodation consists of a waiting-hall which is badly heated and without adequate ventilation, with an ordinary wooden floor. On one side there are four small rooms, boxes would be a better description of these miserably inadequate spaces, for they are not rooms at all in the modern out-patient sense, all of which are badly lighted, and one of which is not available on busy days for much of the time, owing to its being used as an inquiry office for the issue of tickets to new patients. Not only are the side rooms wretchedly small, but in the one where the surgical cases are seen by the Resident Surgical Officer and House Surgeon, and where the Assistant Surgeon has to work when he arrives, there are often six persons: three medical men and three patients, the latter of whom may be of either sex, a condition of affairs which is not only disgraceful but must render it practically impossible for the work to proceed expeditiously or properly at all. Equally grave objections apply to the examination rooms, one of which is used for medical, and one for ophthalmic cases, but ordinary surgical cases are also seen, we understand, in the former room on certain days. To make matters worse, not only is there no proper organisation for the reception and handling of new cases, but owing, we presume, to the inadequate number of the present medical staff, only two days a week, Tuesday and Friday, are given up to out-patients, when the waiting-hall is absolutely packed, and there is often, we were informed, not even standing room for the patients. So bad is the accommodation both structurally and in regard to furniture that it must be impossible for the medical staff to examine any patient in the prone position, the only furniture provided being one wooden bench, and the only examination rooms two small cubicles, one for surgical and one for medical cases, which are incompletely separated, permitting conversations to be overheard, and rendering it impossible to avoid a male patient in one room and a female in the other from undergoing examination at the same time. It is but the truth to declare that no satisfactory work can be done in the out-patient department as it exists to-day, owing to the incessant noise which prevails throughout the rooms, nor is it possible to deal efficiently with the great numbers which the staff have to see, without being provided with those conveniences without which no hospital committee is justified in encouraging the attendance of out-patients for treatment at a modern hospital. We hope that the working men and other governors of this hospital will make it their business to visit the out-patient department while the work is proceeding on a Tuesday or Friday, without delay, for words would fail to depict with sufficient force the cruelty and the wrong which the existing state of affairs inflicts upon the medical staff, and upon the patients who are entitled to special consideration, owing to the magnificent support this hospital receives from the workmen of the Potteries.

The Worst Casualty Rooms in England.

Shameful as the out-patient department and its arrangements are, those of the surgical and casualty rooms are, if possible, infinitely worse. They exhibit an absence of conveniences, a want of privacy and decency, and a callous disregard for what is due to the reputation of the hospital and the feelings and rights of the patients, which we feel justified in declaring is to be found in no other medical institution in the United Kingdom at the present day. In the surgery and casualty rooms a very large number of operations have to be performed. There is no operation theatre, no recovery room, and all patients who have undergone operation are perforce herded together in one small room. The noises of the patients who are being anæsthetised can be heard by those who are waiting to undergo operation. Owing to a want of accommodation gynæcological cases cannot be examined in the ordinary course, but they have to be seen in the surgery, and often have to wait a very long time until the out-patients' operations are completed. We feel it to be a public duty to state plainly the facts as they have been brought to our notice, and as any governor may find them who will go to the Hospital on a busy day and show sufficient grit to watch the work and see with his own eyes exactly what is going on.

Many Defects in Other Departments.

Pathology is one of the most important branches of medical work. At this Hospital the pathologist has only a dark room in the basement, which is so insanitary and devoid of necessary and proper fittings, that it is a source of danger to himself and to others. It is practically impossible to do scientific work in the existing conditions; but in spite of every difficulty good work has been attempted, we were glad to learn, and the Hospital has been saved an annual expenditure of from £30 to £40 a year.

As to the general condition of the Hospital, new bedsteads and feather pillows are an urgent necessity, the majority of the former being ancient, unsafe, unsuitable and impossible if the patients are to be nursed under modern conditions. New lockers might be supplied with advantage, and steps should be taken to establish a proper system of hot-air baths in the medical wards, for the present plan of depending upon a spirit lamp, which involves a constant danger from fire and has often resulted in accidents, is necessarily most inadequate.

The condition of other departments not generally seen is unsatisfactory. The laundry needs entire reconstruction and re-organisation, so as to provide for the reception of soiled linen, and the adequate and proper drying of the clothes. The space available is too limited, and the whole place needs to be taken in hand and brought up-to-date. The boilers are inadequate or defective; the out-buildings, especially the engineer's room, the joiner's shop, and the upholsterer's room, are badly placed, or inadequate, or unusable. The electrical department so-called is not a credit to the Hospital, and should never have been commenced under the conditions in which the work has to be carried on at the present time. The dispensary, especially in regard to the out-patient branch, requires many improvements.

The Resident Staff.

Generous attention should be shown to the comforts of the residents. We would suggest the provision of a billiard-room or some other form of recreation for the resident medical staff, who have to work very hard, and are practically isolated from the outside world by the situation of the hospital and the limited time they can spare from their duties. This is especially the case in the winter months. The discipline and efficiency of the establishment would be promoted by the provision of special rooms for the porters when on and off duty. In the absence of such provision it must be a difficult matter for the management to keep them apart from the female servants, as is invariably done in the best managed hospitals.

A Question of Finance.

We quite understand that the first objection which may occur to the recommendations which we have ventured to make will be the difficulty of providing funds. The institution has some £60,000 invested, and it is therefore in a position to face the necessary expenditure. But an appeal setting forth the facts and asking for funds to bring the hospital thoroughly up-to-date, would, we are confident, speedily produce the relatively small sum required. The hospital has ready to hand a reserve fund which, if properly worked in a business community like that to the needs of which this hospital ministers, ought to yield abundant fruit. The privileges granted to governors and subscribers at the present time are far too great, if regard is to be had to the cost of treating an in-patient and out-patient in the present day. We would suggest that the actual cost of each in-patient and out-patient should be worked out, and that the fact that the present scale of tickets is neither good business nor good charity should be driven home. This has been done successfully in the case of other provincial hospitals, and it is a point which should engage the immediate attention of the governors of the North Staffordshire Infirmary. In any case, the need for immediate reform and reconstruction is urgent; whilst the financial difficulty is one of moderate proportions, which business men may properly face and should readily overcome. In these circumstances, seeing that the reputation of the hospital, its committee and governors is at stake, we venture to hope that immediate steps may be taken to face the position here presented, and to deal with it effectually.

